# Supplementary material for: The relational association between multiple sexual partners and HIV testing on cervical cancer screening among women of reproductive age in Ghana: A national population-based study
Source: PLoS One. 2026 Mar 16;21(3):e0344086. doi: 10.1371/journal.pone.0344086 (PMC12991245; doi:10.1371/journal.pone.0344086)
Supplement: S1 File — (DOCX) [file pone.0344086.s001.docx]

**Forest plots for multivariable results**
